# A retrospective cohort study of supervised and home baked and/or neat hen's egg challenge for pediatric IgE hen's egg allergy

**DOI:** 10.1111/pai.70268

**Published:** 2026-01-20

**Authors:** Caitlin Pollock, Benjamin James Talks, Julie Pentland, Emily Walton, Carina Venter, Louise J. Michaelis

**Affiliations:** ^1^ Newcastle Upon Tyne Hospitals NHS Foundation Trust Newcastle upon Tyne UK; ^2^ Newcastle University Biosciences Institute Newcastle upon Tyne UK; ^3^ Great North Children's Hospital, Newcastle Upon Tyne Hospitals NHS Foundation Trust Newcastle upon Tyne UK; ^4^ Section of Allergy & Immunology, School of Medicine University of Colorado Denver, Children's Hospital Colorado, Anschutz Medical Campus Aurora Colorado USA; ^5^ Newcastle University Population Health Sciences Institute Newcastle upon Tyne UK

**Keywords:** food allergy, food challenge, hen's egg allergy, IgE‐mediated allergy, pediatric

To the Editor(s),

IgE‐mediated food allergy and anaphylaxis are a growing burden on healthcare providers. Consequent highly restrictive diets are associated with faltering growth, further sensitization and allergy, and poor quality of life.^1^ In‐hospital oral food challenge is the gold standard for reintroducing allergenic foods, referred to as “supervised introduction” (SI). Home introduction (HI) is an alternative strategy that has been recommended for IgE‐mediated hen's egg allergy in the British Society of Allergy and Clinical Immunology (BSACI) 2021 guideline.^2^ A HI challenge involves graded reintroduction of hen's egg protein into the diet under parental supervision over days to weeks via a “food ladder” with increasing allergenicity at each “stage”.^2^ HI has the potential to improve patients' healthcare experience as food is reintroduced in a familiar environment whilst reducing the burden on healthcare providers. There have been limited studies on the efficacy of HI or patient factors associated with HI success, representing an area in need of further research.^2^, ^3^

To consider this unmet need, we carried out a single‐center retrospective cohort study to review outcomes from a total of 205 oral hen's egg challenges (baked and neat) performed by 197 children with IgE mediated hen's egg allergy over a 12‐month period (2021). This diagnosis is confirmed by a clinical history of an IgE‐mediated reaction to hen's egg together with supporting skin prick test (SPT), specific IgE results (SpIgE) or Component Resolved Diagnostics (CRD) results. The primary outcome was challenge outcome for HI versus SI. Secondary outcomes included subsequent dietary inclusion, adverse reactions to challenge and risk factors related to challenge failure (regardless of setting or egg type). When children had undergone multiple challenges within the same year, only their first challenge result was included in subsequent analysis (131 HI and 66 SI). For risk factor analysis, a total of 181 challenges (excluding those with inconclusive or unknown outcomes) were compared, 127 SI and 55 HI.

HI was first adopted in our department with the switch to remote consultations during the COVID‐19 pandemic with limited SI availability. Additionally, parents declined face‐to‐face appointments, opting to undertake a challenge at home as preference. These children were provided with a food ladder (either baked or neat hen's egg) to follow. We took the opportunity to review these patients, comparing with the SI cohort along with risk factor analysis to inform future guidance and protocols for HI.

Children were allocated SI following multi‐disciplinary team discussion. These children attended the day‐case ward for a nurse‐supervised challenge following National and European protocols.^4^, ^5^ As such, neat hen's egg challenges were only offered to children who tolerated baked hen's egg 3–4 times a week, as per guidance.^4^, ^5^

HI (81.7% (49/60)) was successfully implemented with a comparable pass rate (no reaction), as SI (81.7% (107/131)) with 6 children having an unknown result from HI. Children undergoing SI were typically older at challenge (39.0 ± 57.0 months for SI, 27.5 ± 39.0 months for HI), potentially due to more severe or complex allergic disease, and long SI waiting lists. The BSACI recommends children be older than 12 months for HI. In this study, 19 children, aged less than 12 months, underwent HI with only 1 (5.0%) failure.^2^ The safety of HI in this age group is further supported by a large retrospective study of 675 children, in which severe challenge reactions were rarest in children younger than 12 months.^6^ Indeed, the “Enquiring About Tolerance Study” further advocates the need for early introduction of well‐cooked hen's egg (before 6 months of age), supported by a recent retrospective study recommending low dose challenges in children <12 months.^7^, ^8^

Importantly, HI proved safe, with only 6/66 (9.1%) children suffering an adverse reaction: 4/6 (66.7%) had skin reactions, and 2/6 (33.3%) had skin and gastrointestinal reactions. Most of these children (5/6, 83.3%) were given antihistamines, no child required hospital admission nor experienced anaphylaxis.

All children who underwent HI reintroduced hen's egg into their diet compared to only 41/49 (83.7%) children who passed SI during the study period with unknown inclusion for 52/107 of SI cohort This may reflect increased parental confidence, having already reintroduced the allergen in their home environment. Subsequent follow‐up, reviewed in 2023/24, highlighted that only 30/107 (28.0%) of SI did not reintroduce hen's egg into the diet. The reasons given for noninclusion aged >11 was persistent patient stress/anxiety and eczema flare (but no IgE symptoms), whilst those <5 years of age was a result of parental anxiety and child food refusal linked to taste/texture.

It is an ongoing clinical dilemma to predict high‐risk children for failing a food challenge. We compared passed (*n* = 156; 86.2%) and failed (*n* = 25; 13.8%) hen's egg challenge of any type (HI or SI) to look for variables that were predictive of challenge outcome (Table 1). The BSACI guideline defines high‐risk children as those with uncontrolled atopic asthma, severe multisystem allergy, or severe allergic reaction symptoms.^2^

**TABLE 1:** Baseline demographics, past medical history, index reaction, egg type, and investigation results for 181 children undergoing home introduction and supervised introduction, comparing those who passed and failed their challenge.

Outcome of introduction	Pass				Fail				*p* Values
Location of introduction	Supervised		Home		Supervised		Home		
	*N*	median ± IQR/*n* (%)	*N*	median ± IQR/*n* (%)	*N*	median ± IQR/*n* (%)	*N*	median ± IQR/*n* (%)	
Demographics									
Age at introduction, months	107	46.0 ± 55.5	49	32.0 ± 31.8	19	63.0 ± 82.5	6	25.2 ± 8.6	.31
Sex, female	107	39 (34.6)	49	22 (44.9)	19	8 (42.1)	6	3 (50.0)	.71
Ethnicity	107		49		19		6		.19
White		77 (72.0)		35 (71.4)		12 (63.2)		6 (100.0)	
White—Other		3 (2.8)		0 (0.0)		0 (0.0)		0 (0.0)	
Mixed—White/Black Caribbean		0 (0.0)		0 (0.0)		0 (0.0)		0 (0.0)	
Mixed—White/Asian		3 (2.8)		2 (4.1)		0 (0.0)		0 (0.0)	
Asian and British Asian—Indian		0 (0.0)		1 (2.0)		1 (5.3)		0 (0.0)	
Asian and British Asian—Pakistani		5 (4.7)		3 (6.1)		0 (0.0)		0 (0.0)	
Asian and British Asian—Bangladeshi		3 (2.8)		0 (0.0)		2 (10.5)		0 (0.0)	
Chinese		0 (0.0)		0 (0.0)		1 (5.3)		0 (0.0)	
Black and Black British—African		0 (0.0)		1 (2.0)		1 (5.3)		0 (0.0)	
Mixed—Other		0 (0.0)		1 (2.0)		0 (0.0)		0 (0.0)	
Other		14 (13.1)		6 (12.2)		2 (10.5)		0 (0.0)	
Past medical history									
Atopic dermatitis	107	89 (83.2)	49	41 (83.7)	19	16 (84.2)	6	6 (100.0)	.77
Allergic rhinitis	107	32 (29.9)	49	5 (10.2)	19	12 (63.2)	6	1 (16.7)	.**01**
Atopic asthma	107	13 (12.2)	49	3 (6.1)	19	6 (31.6)	6	1 (16.7)	.03
Uncontrolled atopy	91	12 (13.1)	45	2 (4.4)	17	3 (17.7)	6	0 (0.0)	.98
Additional food allergy	107		49		19		6		.05
One additional		33 (30.1)		10 (20.4)		7 (36.8)		2 (33.3)	
More than one		31 (29.0)		11 (22.4)		8 (42.1)		3 (50.0)	
Previous anaphylaxis to egg protein	76	7 (9.2)	37	1 (2.7)	16	0 (0.0)	6	0 (0.0)	.36
Dietician input	107	50 (46.7)	49	24 (49.0)	19	13 (68.4)	6	5 (83.3)	.04
Index reaction									
Type of egg	103		48		17		6		.92
Baked		12 (11.7)		3 (6.3)		2 (11.8)		0 (0.0)	
Neat		91 (88.3)		42 (87.5)		15 (88.2)		6 (100.0)	
Symptoms	107		49		19		6		.21
Respiratory symptoms^a^		16 (15.0)		5 (10.9)		5 (26.3)		0 (0.0)	
Skin symptoms only^b^		67 (63.2)		36 (73.5)		9 (47.4)		5 (83.3)	
Plus atopic dermatitis flare		10 (9.4)		6 (12.2)		0 (0.0)		0 (0.0)	
Plus gastrointestinal symptoms^c^		31 (29.0)		9 (18.4)		7 (36.8)		1 (16.7)	
Investigations									
Skin prick test (mm)	97	4.0 ± 3.0	42	2.5 ± 5.0	19	6.0 ± 2.5	4	4.5 ± 2.0	.**01**
Specific IgE—egg (KAU/L)	80	2.7 ± 6.5	26	1.4 ± 5.25	17	13.3 ± 25.5	3	0.8 ± 2.1	.**01**
Total IgE (KAU/L)	21	281 ± 2071	7	101.0 ± 574.9	6	461.5 ± 961.3	0	–	.74
CRD—Ovomucoid (ISU‐E)	61	0.7 ± 2.6	20	0.0 ± 1.2	15	2.0 ± 7.6	3	0.0 ± 0.0	.37
CRD—Ovalbumin (ISU‐E)	13	1.8 ± 3.3	5	1.1 ± 1.2	4	21.3 ± 10.1	0	–	.06
Reintroduction									
Type of egg	107		49		19		6		.23
Baked		42 (39.2)		28 (57.1)		12 (63.2)		3 (50.0)	
Neat		65 (60.8)		21 (42.9)		7 (36.8)		3 (50.0)	

*Note*: Bonferroni corrected significance level for each category of multiple comparisons: demographics *p* < .02, past medical history <0.01, and investigations <0.01. Significant *p* values highlighted in bold.

Abbreviations: CRD, component‐resolved diagnosis; ISU‐E, ImmunoCAP standardized units for immunoglobulin E; IQR, interquartile range.

^a^ Wheeze, cough, difficulty breathing associated with anaphylaxis.

^b^ IgE skin symptoms; urticaria, erythema, angioedema and pruritis.

^c^ Vomiting, constipation, or loose stools (non‐IgE symptoms).

In this study, co‐existing allergic rhinitis and atopic asthma were associated with challenge failure, in keeping with their position as endpoints of the “atopic march”.^9^ Of those children with asthma, 8/26 (30.8%) had poor control with 2/8 (20%) failing challenge, highlighting the importance of asthma control before challenge. Additionally, a higher rate of allergic rhinitis was seen in children who failed, 13/25 (52.0%) versus only 37/156 (23.7%) who passed. Therefore, we recommend avoiding HI in children with allergic rhinitis and atopic asthma. Interestingly, rates of atopic dermatitis were comparable in pass and fail cohorts with an incidence of 86.1% (130/156) and 88.0% (22/25), respectively.

Previous severe reactions to hen's egg were documented for 29/181 (16.0%) children: 18/23 (78.3%) passed SI and 6/6 (100.0%) passed HI. This demonstrates the potential for HI for children with no recent severe reactions within 6 months.

Of the baseline laboratory investigations performed, only SPT (whole hen's egg) and SpIgE (whole hen's egg) differed significantly between children who had passed and failedtheir challenge. Local calculated cutoff values for SPT and SpIgE (using area under the curve receiver‐operating‐characteristic analysis to maximize sensitivity and specificity) were SPT 4.5 mm and SpIgE 10.75 KAU/L, see Figure 1. This is markedly lower than the BSACI SPT recommendation of >13 mm, likely due to the use of different testing solutions, and highlighting the need for local SPT cutoffs depending on test solution used.^2^ Although routine testing of CRD is not recommended in the BSACI guideline, aa prospective study reports ovomucoid ISU‐E > 11.0 are associated with challenge failure.^2^, ^10^ In our study, of the 103 children who had CRD ovomucoid tested, 6 (5.8%) had a result >11.0 ISU‐E, all of whom underwent SI, and 3/6 (50.0%) failed.

**FIGURE 1:**
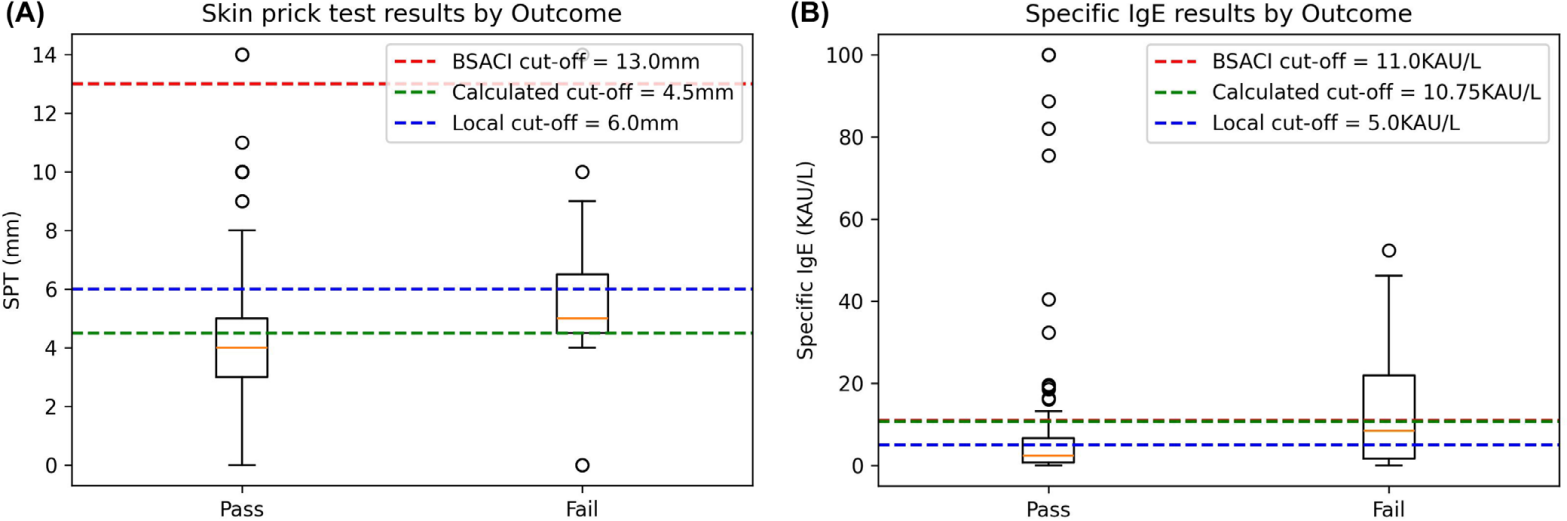
Investigation results from the pass and fail cohorts with cutoffs demonstrated. (A) Skin prick test and (B) Specific IgE were significantly lower in children that passed this challenge, *p* = .01 for both.

In summary, HI is safe and feasible for challenge and subsequent introduction of hen's egg protein in low‐risk children with IgE‐mediated allergy. HI supports service provision, as HI has the potential to avoid prolonged waiting lists and prevent restrictive diets. However, patient selection is critical. We identified high‐risk children as those with previous severe reactions to hen's egg, uncontrolled atopic disease, older age group (>16 years), SPT >4.5 mm, or SpIgE >10.75 KAU/L. Clinical practice varies at the local and national level. Therefore, standardized HI protocols are needed to facilitate safe and effective HI, develop HI services, and improve comparability of cohorts.

## AUTHOR CONTRIBUTIONS

**Caitlin Pollock:** Data curation; investigation; formal analysis; methodology; writing – original draft. **Benjamin James Talks:** Writing – review and editing; supervision. **Julie Pentland:** Data curation; supervision. **Emily Walton:** Data curation. **Carina Venter:** Writing – review and editing. **Louise J. Michaelis:** Conceptualization; supervision; resources; writing – review and editing.

## CONFLICT OF INTEREST STATEMENT

None of the authors have a conflict of interest to disclose in relation to this work. Dr. Michaelis has previous national commercial clinical trials with associated Advisory Boards / Lectures with Danone, ALK and Regeneron. Dr. Michaelis is Vice President for Services for the British Society of Allergy and Immunology, United Kingdom. Dr. Venter reports grants from Mead Johnson Nutrition and personal fees from Mead Johnson Nutrition, Nestle Nutrition Institute, Danone, Abbott Nutrition, and Novalac outside the submitted work.

## ETHICS STATEMENT

This retrospective cohort study was registered with the Clinical Effectiveness Register of Newcastle Upon Tyne Hospitals NHS Foundation Trust (project number: 13495). Since this was a retrospective study reviewing routine medical notes, individual patient consent was not obtained and would not be practicable to obtain.
